# Reciprocal grafting separates the roles of the root and shoot in zinc hyperaccumulation in *Thlaspi caerulescens*

**DOI:** 10.1111/j.1469-8137.2009.02969.x

**Published:** 2009-10

**Authors:** Marcelo de A Guimarães, Jeffery L Gustin, David E Salt

**Affiliations:** Department of Horticulture and Landscape Architecture, Purdue UniversityWest Lafayette, IN 47906, USA

**Keywords:** chlorophyll, grafting, hyperaccumulation, inductively coupled plasma mass spectroscopy (ICP-MS), *Thlaspi caerulescens*, *Thlaspi perfoliatum*, zinc (Zn)

## Abstract

- The extreme phenotype of zinc (Zn) hyperaccumulation, which is found in several Brassicaceae species, is determined by mechanisms that promote elevated Zn tolerance and high Zn accumulation in shoots.
- We used reciprocal grafting between a Zn hyperaccumulator, *Thlaspi caerulescens*, and a Zn nonaccumulator, *Thlaspi perfoliatum*, to determine the relative importance of roots and shoots in Zn hyperaccumulation and hypertolerance.
- Leaves from plants with a *T. perfoliatum* rootstock and a *T. caerulescens* shoot scion did not hyperaccumulate Zn, whereas plants with a *T. caerulescens* rootstock and a *T. perfoliatum* shoot scion did hyperaccumulate Zn. However, although leaves from plants with a *T. caerulescens* rootstock and a *T. perfoliatum* shoot scion hyperaccumulated Zn, at high Zn loads these leaves showed significant symptoms of Zn toxicity, unlike leaves of self grafted *T. caerulescens*.
- Hyperaccumulation of Zn in leaves of the hyperaccumulator *T. caerulescens* is pri-marily dictated by root processes. Further, the mechanisms controlling Zn hypertolerance in the hyperaccumulator *T. caerulescens* are driven primarily by shoot processes.

## Introduction

Zinc (Zn) is an essential micronutrient required for many plant processes, but in excess Zn can be toxic. Plants therefore regulate Zn accumulation to provide the Zn required for essential processes, yet protect against toxicity resulting from over-accumulation. A unique group of plant species have evolved the ability to hyperaccumulate Zn to up to 30 000 µg g^−1^ of their shoot dry biomass when growing in their native habitat (reviewed in Baker *et al*., 2000). This is 10–100-fold higher than for other plants growing in the same location. The mechanisms governing the elevated Zn accumulation and tolerance in two hyperaccumulators, *Thlaspi caerulescens* and *Arabidopsis halleri*, are under independent genetic control in each plant species, and governed by a limited number of genes (reviewed in Verbruggen *et al*., 2009).

It is now clear that the enhanced shoot Zn accumulation in *A. halleri* is primarily driven by the constitutively elevated expression of *AhHMA4* (HMA, Heavy Metal ATPase ), which encodes a Zn transporter involved in releasing Zn from the root symplast into the xylem vessels for translocation of Zn to the shoot (Courbot *et al*., 2007; Willems *et al*., 2007; Hanikenne *et al*., 2008). This elevated flux of Zn to the shoot in *A. halleri*, driven by HMA4, also signals increased expression of plasma membrane Zn transporters which leads to increased Zn uptake by the root (Hanikenne *et al*., 2008). It is likely that HMA4 also plays a similar role in *T. caerulescens* (Bernard *et al*., 2004).

There is now growing evidence that constituently high shoot expression of the vacuolar Zn transporter *MTP1* (MTP1, Metal Tolerance Protein1) plays an important role in Zn hypertolerance in the Zn hyperaccumulator *A. halleri* (Becher *et al*., 2004; Dräger *et al*., 2004; Willems *et al*., 2007), and possibly in *T. caerulescens* (Assunção *et al*., 2001) and *Thlaspi goesingense* (Persans *et al*., 2001; Gustin *et al*., 2009). Furthermore, it has recently been shown that heterologous high expression of *MTP1*, specifically in shoots of the nonaccumulator *Arabidopsis thaliana*, leads to both elevated Zn tolerance and enhanced expression of Zn transporters in the root and shoot (Gustin *et al*., 2009). By providing an enhanced vacuolar sink for Zn in the shoots of hyperaccumulators, constitutively high expression of *MTP1* both confers enhanced Zn tolerance and initiates a Zn-deficiency signal that activates the expression of Zn transporters in both the shoots and the root.

Together, elevated levels of HMA4 and MTP1 in hyperaccumulators appear to provide a feedback circuit between the root and shoot, which not only increases the uptake and flux of Zn from the soil via the roots to the shoots, but also allows this elevated Zn flux to be funneled into the vacuolar compartment of shoot cells for detoxification and long-term storage.

If correct, this model predicts that Zn hyperaccumulation should be primarily driven by root processes, with only a minor component of Zn accumulation being influenced by the shoot. By contrast, we would predict that Zn hypertolerance should be primarily determined by shoot processes. To directly test this model we carried out a study in which we reciprocally grafted rootstocks and shoot scions from the Zn hyperaccumulator *T. caerulescens* and the nonaccumulator *Thlaspi perfoliatum*. Results from these experiments are fully consistent with this model of Zn hyperaccumulation in which the root is the primary driver of Zn hyperaccumulation and shoot Zn hypertolerance.

## Materials and Methods

### Plant material

Original seeds of the hyperaccumulator *Thlaspi caerulescens* St Félix de Palliéres (Tc) and the nonaccumulator *Thlaspi perfoliatum* Col de Gláize (Tp) were collected from France as described by Peer *et al*. (2003).

### *Thlaspi* grafting

Grafting was performed using a method adapted from Rus *et al*. (2006). Seeds of *T. caerulescens* and *T. perfoliatum* were stratified dry for 5 d at 4°C in the dark. Stratified seeds were surface-sterilized with a brief incubation in 70% EtOH followed by a 5-min treatment with 10% bleach/0.1% Tween20. Sterilized seeds were washed six times with sterile deionized H_2_O, and mixed into a 0.1% agar solution. Seeds were sown onto nutrient agar plates containing 0.5× MS Vitamin Stock (Caisson Laboratories, Inc., North Logan, UT, USA), 12 g l^−1^ agar (Sigma-Aldrich Inc., St Louis, MO, USA), and 3 mg l^−1^ Benonyl (methyl 1-(butylcarbamoyl)-2-benzimidazolecarbamate; Sigma, St Louis, MO, USA) in 100 × 15 mm polystyrene Petri dishes. Seed concentrations were 20 seeds per plate to produce self-grafted plants, and 10 seeds per plate for reciprocally grafted plants. Petri dishes were placed vertically and seeds allowed to germinate in a glasshouse at 23°C day : 18°C night, with a 16 : 8 h day:night cycle. Seven days after sowing, seedlings to be grafted were cut mid-hypocotyl on the agar surface to produce a 90° blunt end, using a 15° Stab Knife (Fine Scientific Tools, North Vancouver, BC, Canada) without collars (Turnbull *et al*., 2002). All grafting manipulations were conducted with a stereomicroscope (Ken-A-Vision, model T-2200, Ken-A-Vision, Inc., Kansas City, MO, USA). Seedlings remained on the plates for 17 d to allow the formation of the graft union.

### Plant growth conditions

Successfully grafted seedlings were transferred directly to moist soil (Premier Promix PGX, Premier Horticulture Inc., Quakertown, PA, USA). Plants were watered once per week with Purdue University Greenhouse fertilizer containing (in mg l^−1^) 200 nitrogen (N), 29 phosphorus (P), 167 potassium (K), 67 calcium (Ca), 30 magnesium (Mg), and micronutrients supplied from a commercial fertilizer formulation (MiracleGro® Excel® 15-5-15 Cal-Mag; The Scotts Co., Marysville, OH, USA) with pH adjusted to the range 5.7–6.0 (Aschenbeck & Eddy, 2004). The plants were grown in the glasshouse environment described in the previous section. After 4 wk, the plants were randomly divided into two blocks, a control block and a Zn treatment block, each containing a representative set of grafted individuals. The control block receive an alternating schedule of tap water and standard glasshouse fertilized water, while the Zn treatment block received the same watering schedule with different Zn(NO_3_)_2_ concentrations (0.5, 1, 2, 4, 8 and 16 mm) added to each fertilizer treatment once at weekly intervals.

### Quantification of shoot Zn, cadmium (Cd) and chlorophyll

One fully expanded leaf (5–10 mg DW) from each plant was harvested and analyzed by inductively coupled plasma mass spectroscopy (ICP-MS) beginning when plants were 4 wk old, and before the first supplemental Zn treatment. Thereafter, leaves were harvested at weekly intervals following each Zn treatment. Harvested leaves were rinsed with 18 MΩ water and placed in Pyrex digestion tubes (16 × 100 mm), and the samples were dried for 24 h at 92°C and weighed after cooling. All samples were digested under open-air conditions using 0.7 ml of concentrated nitric acid (AR Select grade; Mallinckrodt Baker Inc., Phillipsburg, NJ, USA) at 110°C for 4 h, and diluted to 6.0 ml with 18 MΩ water. The samples were analyzed using ICP-MS (Elan DRCe; PerkinElmer, Waltham, MA, USA) for lithium (Li), boron (B), sodium (Na), Mg, P, K, Ca, manganese (Mn), iron (Fe), cobalt (Co), nickel (Ni), copper (Cu), Zn, arsenic (As), selenium (Se), molybdenum (Mo), and Cd. Chlorophyll content was quantified as the average of independent measurements of three fully expanded leaves from each plant, using a chlorophyll meter (Konica Minolta SPAD-502, Osaka, Japan) at the time of harvest.

### Statistics

Data were statistically analyzed using analysis of variance (ANOVA), and tested for significant (*P* ≤ 0.05) treatment differences using Tukey's test.

## Results

### *Thlaspi* grafting

Seeds of both Thlaspi species showed 100% germination. Four weeks after transplantation from plates to soil, nongrafted *T. caerulescens* seedlings had a survival rate of 98% compared with 88% for *T. perfoliatum* (Table 1). Self-grafted plants tended to show higher rates of transplantation survival compared with the reciprocally grafted plants (Table 1). Plants with *T. perfoliatum* shoot scions tended to show higher rates of survival 1 wk after transplant (82 and 60%, respectively, for self-grafted and reciprocally grafted plants) compared with plants with *T. caerulescens* scions (48 and 37% survival, respectively) (Table 1). The trend for higher survival of *T. perfoliatum* compared with *T. caerulescens* scions was also observed 4 wk after transplantation (Table 1). Differences in transplantation efficiency are probably attributable to differences in callus production at the graft union between the two species. We also found that *T. perfoliatum* tissue was easier to cut than *T. caerulescens*. This may have caused more imperfect 90° blunt-end cuts in *T. caerulescens* as compared with *T. perfoliatum*, resulting in reduced graft union formation. With practice, we were able to perform > 30 grafts h^−1^. Approximately 30% of grafted plants developed adventitious roots from the scion. All adventitious roots were removed.

**Table 1:** Rate of successful transplants from plates to soil of grafted and nongrafted *Thlaspi caerulescens* (Tc) and *Thlaspi perfoliatum* (Tp)

Shoot/root	Transplanted	Week 1	Week 4
Tc	100	100	98
Tc/Tc	55	48	38
Tc/Tp	50	37	27
Tp	100	93	88
Tp/Tp	84	82	71
Tp/Tc	65	60	57

Plants are ungrafted *T. caerulescens* (Tc) and *T. perfoliatum* (Tp), self-grafted *T. caerulescens* (Tc/Tc) and *T. perfoliatum* (Tp/Tp), and plants with a *T. caerulescens* shoot scion with a *T. perfoliatum* rootstock (Tc/Tp) and a *T. perfoliatum* shoot scion with a *T. caerulescens* rootstock (Tp/Tc). Data represent the number of surviving seedlings as a percentage of the total number of seedlings for each type (total seedlings for Tc = 40, Tp = 40, Tc/Tc = 42, Tp/Tp = 49, Tc/Tp = 60, Tp/Tc = 65).

### Role of the root in Zn hyperaccumulation

**Fig. 1:**
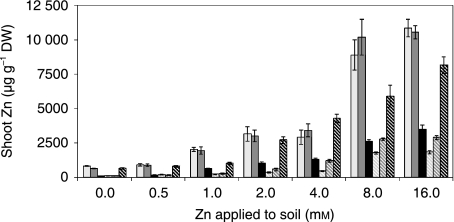
Zinc (Zn) accumulation in shoots of reciprocally grafted plants of the Zn hyperaccumulator *Thlaspi caerulescens* (Tc) and the related nonaccumulator *Thlaspi perfoliatum* (Tp). Bars represent data for grafted and nongrafted plants: light gray, *Thlaspi caerulescens* (Tc); gray, self-grafted *T. caerulescens* (Tc/Tc); dark gray, *T. caerulescens* shoot scion with *T. perfoliatum* rootstock (Tc/Tp); light gray hatched, *T. perfoliatum* (Tp); gray hatched, self-grafted *T. perfoliatum* (Tp/Tp); dark gray hatched, *T. perfoliatum* shoot scion with *T. caerulescens* rootstock (Tp/Tc). Three-week-old plants were treated with increasing concentrations of Zn(NO_3_)_2_ (0, 0.5, 1, 2, 4, 8 and 16 mm) over the course of 7 wk, with a single application of each Zn treatment at weekly intervals. Shoot samples were taken for Zn analysis 1 wk after each treatment, and just before the next treatment. Data represent the mean ± SE. Samples sizes for the experiment were Tc (*n* = 8), Tc/Tc (*n* = 6), Tc/Tp (*n* = 5), Tp (*n* = 7), Tp/Tp (*n* = 7) and Tp/Tc (*n* = 7). ANOVA data are shown in Table 2.

Plant were sampled and metal concentrations measured at the start of the treatment period and subsequently before each treatment. Across all Zn treatments the concentrations of shoot Zn in *T. caerulescens* nongrafted and self-grafted plants were not significantly different (Table 2). A similar result was obtained for *T. perfoliatum* nongrafted and self-grafted individuals (Table 2). This establishes that grafting did not significantly affect shoot Zn accumulation in either species. As expected, nongrafted and self-grafted *T. caerulescens* showed a consistently higher shoot Zn accumulation compared with nongrafted and self-grafted *T. perfoliatum*, and by the end of the experiment, shoots of *T. caerulscence* nongrafted and self-grafted plants had hyperaccumulated > 10 000 µg g^−1^ dry biomass Zn (Table 2,Fig. 1). Strikingly, when *T. caerulescens* was grafted as a scion onto a *T. perfoliatum* rootstock, the *T. caerulescens* shoot no longer accumulated elevated Zn, but rather accumulated Zn similar to *T. perfoliatum* shoots across all Zn treatment levels (Table 2,Fig. 1). Furthermore, when *T. perfoliatum* was grafted as a shoot scion onto a *T. caerulescens* rootstock, Zn accumulation in the *T. perfoliatum* scion was very similar to that of *T. caerulescens* nongrafted or self-grafted individuals at the 0.5, 1, 2 and 4 mm Zn treatment levels (Table 2,Fig. 1). This observation unambiguously establishes that the root of *T. caerulescens* is driving enhanced Zn accumulation in the shoots. However, at 8 and 16 mm Zn treatment the shoot Zn accumulation in plants with *T. perfoliatum* shoot scion and *T. caerulescens* rootstock was reduced compared with *T. caerulescens* (nongrafted or self-grafted) (Table 2,Fig. 1). We propose that this is at least partly a result of the Zn toxicity observed in these plants, as discussed in the below section ‘Role of the shoot in Zn tolerance’.

**Table 2:** Shoot zinc (Zn) accumulation in nongrafted and grafted plants of the Zn hyperaccumulator *Thlaspi caerulescenes* (Tc) and the related nonaccumulator *Thlaspi perfoliatum* (Tp). Plants were treated with increasing concentrations of Zn(NO_3_)_2_ (0, 0.5, 1, 2, 4, 8 and 16 mm) for 7 wk

	Zn applied to the soil (mm)													
Shoot/root	0.0		0.5		1.0		2.0		4.0		8.0		16.0	
Tc	812	a	904	a	2030	a	3143	a	2914	a	8 890	a	10 856	a
Tc/Tc	637	a	873	a	1947	a	3006	a	3411	a	10 193	a	10 551	a
Tc/Tp	82	b	142	b	623	b	1022	b	1306	b	2 770	c	3 489	c
Tp	110	b	174	b	218	c	354	b	446	b	1 760	c	1 824	c
Tp/Tp	106	b	146	b	264	c	575	b	1199	b	2 595	c	2 883	c
Tp/Tc	643	a	792	a	1027	b	2698	a	4274	a	5 889	b	8 161	b

Plants are ungrafted *T. caerulescens* (Tc) and *T. perfoliatum* (Tp), self-grafted *T. caerulescens* (Tc/Tc) and *T. perfoliatum* (Tp/Tp), and plants with a *T. caerulescens* shoot scion with a *T. perfoliatum* rootstock (Tc/Tp) and a *T. perfoliatum* shoot scion with a *T. caerulescens* rootstock (Tp/Tc). Different letters denote significant differences between means for grafted and nongrafted plants under the same treatment (ANOVA test, *P* ≤ 0.05). Samples sizes for the experiment were Tc (*n* = 8), Tc/Tc (*n* = 6), Tc/Tp (*n* = 5), Tp (*n* = 7), Tp/Tp (*n* = 7) and Tp/Tc (*n* = 7).

### Role of the root in cadmium accumulation

**Fig. 2:**
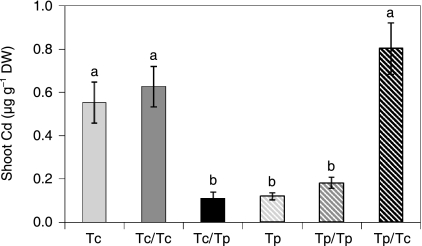
Cadmium (Cd) accumulation in reciprocally grafted plants of the zinc (Zn) hyperaccumulator *Thlaspi caerulescens* (Tc) and the related nonaccumulator *Thlaspi perfoliatum* (Tp) grown for 10 wk without the addition of supplemental Zn. Bars represent data for grafted and nongrafted plants: light gray, *Thlaspi caerulescens* (Tc); gray, self-grafted *T. caerulescens* (Tc/Tc); dark gray, *T. caerulescens* shoot scion with *T. perfoliatum* rootstock (Tc/Tp); light gray hatched, *T. perfoliatum* (Tp); gray hatched, self-grafted *T. perfoliatum* (Tp/Tp); dark gray hatched, *T. perfoliatum* shoot scion with *T. caerulescens* rootstock (Tp/Tc). Data are mean ± SE. Samples sizes for the experiment were Tc (*n* = 8), Tc/Tc (*n* = 6), Tc/Tp (*n* = 5), Tp (*n* = 7), Tp/Tp (*n* = 7) and Tp/Tc (*n* = 7). Different letters denote significant differences between means for grafted and nongrafted plants under the same treatment (ANOVA test, *P* ≤ 0.05).

The accession of *T. caerulescens* used in this study (St Félix de Palliéres) is also well documented to hyperaccumulator Cd in its native habitat (Robinson *et al*., 1998; Reeves *et al*., 2001). Trace levels of Cd were present in the soil mix used in these experiments, which allowed us to examine the effects of grafting on Cd accumulation. As expected, Cd concentrations in the shoots of *T. caerulescens* (nongrafted and self-grafted) were elevated compared with *T. perfoliatum* (nongrafted and self-grafted) (Fig. 2). Similar to findings for Zn, the elevated shoot Cd observed in *T. caerulescens* was also driven primarily by the root, as plants with a *T. caerulescens* rootstock and a *T. perfoliatum* shoot scion accumulated Cd to the same levels observed in *T. caerulescens* (nongrafted and self-grafted). Further, the hyperaccumulator shoot appears not to influence Cd accumulation, as plants with a *T. caerulescens* shoot scion and a *T. perfoliatum* rootstock accumulated Cd in shoots to the level observed in *T. perfoliatum* (nongrafted and self-grafted).

### Role of the shoot in Zn tolerance

**Fig. 3:**
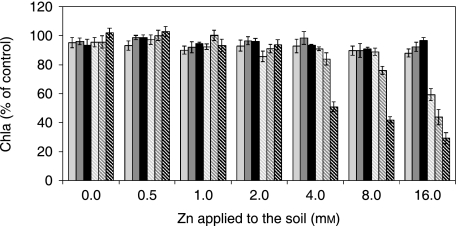
Chlorophyll a content of reciprocally grafted plants of the zinc (Zn) hyperaccumulator *Thlaspi caerulescens* (Tc) and the related nonaccumulator *Thlaspi perfoliatum* (Tp). Bars represent data for grafted and nongrafted plants: light gray, *Thlaspi caerulescens* (Tc); gray, self-grafted *T. caerulescens* (Tc/Tc); dark gray, *T. caerulescens* shoot scion with *T. perfoliatum* rootstock (Tc/Tp); light gray hatched, *T. perfoliatum* (Tp); gray hatched, self-grafted *T. perfoliatum* (Tp/Tp); dark gray hatched, *T. perfoliatum* shoot scion with *T. caerulescens* rootstock (Tp/Tc). Three-week-old plants were treated with increasing concentrations of Zn(NO_3_)_2_ (0, 0.5, 1, 2, 4, 8 and 16 mm) over the course of 7 wk, with a single application of each Zn treatment at weekly intervals. Shoot samples were taken for chlorophyll analysis 1 wk after each treatment, and just before the next treatment. Data represent mean ± SE of leaf chlorophyll content as a percentage of the average chlorophyll content in plants of the same age untreated with Zn. Samples sizes for the experiment were Tc (*n* = 8), Tc/Tc (*n* = 6), Tc/Tp (*n* = 5), Tp (*n* = 7), Tp/Tp (*n* = 7) and Tp/Tc (*n* = 7).

**Fig. 4:**
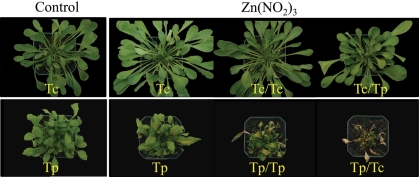
Visible symptoms of zinc (Zn) toxicity in reciprocally grafted plants of the Zn hyperaccumulator *Thlaspi caerulescens* and the related nonaccumulator *Thlaspi perfoliatum*. Represented plants are: ungrafted *T. caerulescens* (Tc) and *T. perfoliatum* (Tp); self-grafted *T. caerulescens* (Tc/Tc) and *T. perfoliatum* (Tp/Tp); and plants with *T. caerulescens* shoot scion with *T. perfoliatum* rootstock (Tc/Tp) and *T. perfoliatum* shoot scion with *T. caerulescens* rootstock (Tp/Tc). Three-week-old plants were treated with increasing concentrations of Zn(NO_3_)_2_ (0, 0.5, 1, 2, 4, 8 and 16 mm) over the course of 7 wk, with a single application of each Zn treatment at weekly intervals. Controls were untreated. At the end of the 7-wk treatment period images were taken.

Previous research has established chlorophyll content as a good marker with which to evaluate Zn toxicity when comparing Zn hyperaccumulator with nonaccumulator plants (Lasat *et al*., 1996). Based on this, chlorophyll content was used to evaluate Zn toxicity in the grafted and nongrafted hyperaccumulator *T. caerulescens* and nonaccumulator *T. perfoliatum*. Both nongrafted and self-grafted *T. caerulescens* showed no reduction in leaf chlorophyll or visible symptom of Zn toxicity across all Zn treatments (Figs 3, 4), demonstrating this plant's Zn hypertolerance. However, as expected for a Zn nonaccumulator, nongrafted and self-grafted *T. perfoliatum* showed significant reductions in chlorophyll and clear visible symptoms of Zn toxicity, particularly at the highest Zn treatment of 16 mm (Figs 3, 4). Furthermore, when *T. perfoliatum* was grafted as a shoot scion onto a *T. caerulescens* rootstock, the chlorophyll content of the leaves was reduced at lower concentrations and to a larger extent as compared with nongrafted or self-grafted *T. perfoliatum* (Fig. 3), and visible symptoms of Zn toxicity started appearing after the 4 mm Zn treatment period. We interpret this increased toxicity in the shoot as reflecting the increased shoot Zn that these plants accumulate compared with nongrafted or self-grafted *T. perfoliatum* (Table 2,Fig. 1). Plants with a *T. perfoliatum* rootstock grafted to a *T. caerulescens* shoot scion showed no symptoms of Zn toxicity, similar to nongrafted and self-grafted *T. caerulescens* (Figs 3, 4). These observations indicate that Zn hypertolerance in *T. caerulescens* is conferred predominantly by the shoot, with approximately equivalent Zn tolerance being observed in plants with *T. caerulescens* shoots regardless of the genotype of the roots.

## Discussion

Here we present evidence that the mechanisms governing enhanced shoot Zn accumulation and tolerance in the Zn hyperaccumulator *T. caerulescens* are driven by the root and shoot, respectively. These observations are consistent with the model that constitutively elevated expression of *HMA4* in roots and that of *MTP1* in shoots interact to confer enhanced Zn uptake, xylem loading, shoot accumulation and tolerance in *A. halleri* and *T. caerulescens* (Becher *et al*., 2004; Dräger *et al*., 2004; Willems *et al*., 2007; Hanikenne *et al*., 2008; Gustin *et al*., 2009).

Our grafting studies show that the *T. caerulescens* hyperaccumulator root has an enhanced capacity to uptake and load Zn for transport to the shoot. Furthermore, this function is essentially independent of the shoot, and sufficient to drive the elevated Zn accumulation observed in the hyperaccumulator. This is consistent with the previous observation that roots of *T. caerulescens* have an elevated rate of Zn uptake (Lasat *et al*., 1996; Pence *et al*., 2000). Recently, Hanikenne *et al*. (2008) established that the elevated expression of *HMA4*, encoding a protein involved in loading Zn into the xylem for translocation to the shoot, is required for Zn hyperaccumulation in *A. halleri*. Given the critical role HMA4 plays in enhanced Zn translocation in *A. halleri*, we propose that this protein is also likely to be the factor that drives the root-based Zn hyperaccumulation we have revealed in *T. caerulescens* through our grafting experiments. This conclusion is supported by the fact that *HMA4* expression is known to be elevated in *T. caerulescens* roots compared with roots of the nonaccumulator *A. thaliana* (Bernard *et al*., 2004). Previous studies have established that the ZIP (ZIP, Zn-regulated transporter (ZRT) Fe-regulated transport (IRT)-like proteins) family members *ZIP3*, *ZIP4*, *ZIP9*, and *ZIP10* are highly expressed in the roots of *T. caerulescens* compared with nonaccumulator species (Pence *et al*., 2000; van de Mortel *et al*., 2006). Such observations are also consistent with elevated *HMA4* expression activating Zn uptake in *T. caerulescens* roots, as observed in *A. halleri* (Hanikenne *et al*., 2008).

Various populations of *T. caerulescens* growing in southern France, including the St Félix de Palliéres population used in this study, show shoot Cd accumulation greater than 1000 µg g^−1^, when growing in their native habitat (Robinson *et al*., 1998; Reeves *et al*., 2001). Because of the trace levels of Cd present in the soils used in the experiments described here, the absolute concentrations of shoot Cd observed in *T. caerulescens* were low compared with concentrations observed in plants growing in their native habitat. However, enhanced accumulation of Cd in *T. caerulescens*, compared with the nonaccumulator *T. perfoliatum*, was clearly observed. Further, this enhanced ability to accumulate Cd is driven primarily by the root, in a similar manner to that observed for Zn in these plants. Whether the enhanced Cd and Zn accumulation observed in *T. caerulescens* is driven by the same root-based molecular mechanism is still being debated (reviewed in Verbruggen *et al*., 2009).

With the grafting experiments described here we have established that the mechanism that controls Zn hypertolerance in the hyperaccumulator *T. caerulescens* is determined primarily by the shoot. Grafted plants containing a *T. caerulescens* shoot scion on a *T. perfoliatum* rootstock showed the same level of Zn tolerance as *T. caerulescens*, even though *T. perfoliatum* plants (nongrafted or self-grafted) are sensitive to elevated Zn. The majority of Zn in *T. caerulescens* shoots accumulates in the vacuoles of epidermal and mesophyll cells (Küpper *et al*., 1999, 2000). Therefore, efficient transport of Zn into the vacuole is likely to be an important factor for storage of the accumulated Zn in hyperaccumulator shoots. The vacuolar localized Zn transporter MTP1 has been suggested to play an important role in this vacuolar localization (Assunção *et al*., 2001; Persans *et al*., 2001; Becher *et al*., 2004; Dräger *et al*., 2004; Willems *et al*., 2007; Gustin *et al*., 2009). Recently, high heterologous expression of *MTP1*, specifically in *A. thaliana* shoots, has been shown to be sufficient to confer elevated Zn tolerance (Gustin *et al*., 2009). These data support our suggestion that the shoot-based Zn tolerance mechanism observed in *T. caerulescens* is driven, at least in part, by the elevated *MTP1* expression observed in shoots of the hyperaccumulator.

Direct access to roots of grafted plants would allow further characterization of the role of roots in Zn hyperaccumulation and tolerance. However, our attempts to transfer grafted *Thlaspi* seedlings to hydroponic culture were unsuccessful because of the low survival rate of transferred self-grafted and reciprocally grafted individuals. Poor survival after transfer was apparently attributable to grafting, as nongrafted *T. caerulescens* and *T. perfoliatum* seedlings could be readily transferred successfully. Inability to recover sufficient numbers of grafted plants in hydroponic culture limited our ability to directly assess root physiology. Nevertheless, we feel that our conclusions regarding the role of roots in Zn hyperaccumulation are well supported by measurements of aerial tissue of grafted plants.

In conclusion, the experiments conducted show that Zn hypertolerance of *T. caerulescens* is primarily driven by mechanisms localized in the shoot, with no major dependence on the genotype of the root. Furthermore, the increased root-to-shoot Zn flux, required for Zn hyperaccumulation, is primarily driven by the roots of the hyperaccumulator, and is predominantly independent of the shoot.
